# Using Spin-Coated Silver Nanoparticles/Zinc Oxide Thin Films to Improve the Efficiency of GaInP/(In)GaAs/Ge Solar Cells

**DOI:** 10.3390/ma11061020

**Published:** 2018-06-15

**Authors:** Po-Hsun Lei, I-Jen Chen, Jia-Jan Chen, Po-Chun Yang, Yan-Hua Gong

**Affiliations:** Institute of Electro-Optical and Materials Science, National Formosa University, 64 Wen-Hwa Rd, Hu-Wei, Yun-Lin 623, Taiwan; angelren1010@gmail.com (I.-J.C.); style_rx7@hotmail.com.tw (J.-J.C.); 10676124@gm.nfu.edu.tw (P.-C.Y.); 10676111@gm.nfu.edu.tw (Y.-H.G.)

**Keywords:** Ag NPs/zinc oxide thin film, InGaP/InGaAs/Ge solar cell, spin-coating technology

## Abstract

We synthesized a silver nanoparticle/zinc oxide (Ag NP/ZnO) thin film by using spin-coating technology. The treatment solution for Ag NP/ZnO thin film deposition contained zinc acetate (Zn(CH_3_COO)_2_), sodium hydroxide (NaOH), and silver nitrate (AgNO_3_) aqueous solutions. The crystalline characteristics, surface morphology, content of elements, and reflectivity of the Ag NPs/ZnO thin film at various concentrations of the AgNO_3_ aqueous solution were investigated using X-ray diffraction, scanning electron microscopy, energy-dispersive X-ray spectroscopy, atomic force microscopy, and ultraviolet–visible–near infrared spectrophotometry. The results indicated that the crystalline structure, Ag content, and reflectance of Ag NP/ZnO thin films depended on the AgNO_3_ concentration. Hybrid antireflection coatings (ARCs) composed of SiN_x_ and Ag NPs/ZnO thin films with various AgNO_3_ concentrations were deposited on GaInP/(In)GaAs/Ge solar cells. We propose that the optimal ARC consists of SiN_x_ and Ag NP/ZnO thin films prepared using a treatment solution of 0.0008 M AgNO_3_, 0.007 M Zn(CH_3_COO)_2_, and 1 M NaOH, followed by post-annealing at 200 °C. GaInP/(Al)GaAs/Ge solar cells with the optimal hybrid ARC and SiN_x_ ARC exhibit a conversion efficiency of 34.1% and 30.2% with V_oc_ = 2.39 and 2.4 V, J_sc_ = 16.63 and 15.37 mA/cm^2^, and fill factor = 86.1% and 78.8%.

## 1. Introduction

Multi-junction solar cells (MJSCs) based on III-V compound semiconductors have attracted much attention for space and terrestrial applications because they are composed of inherently tunable and direct bandgap materials, resulting in a high conversion efficiency due to the absorption of a varied solar spectrum [1,2,3]. However, the conversion efficiency of MJSCs depends on not only the absorption but also the intensity of sunlight; consequently, MJSCs exhibit a lack of absorption under one-sun or low-concentration sunlight for terrestrial applications, and this causes low conversion efficiency. To enhance the absorption of the solar spectrum and obtain a high conversion efficiency, several studies have endeavored to optimize the solar cell structures of MJSCs by using various bandgap materials such as graded AlGaInP solar cells, Ga(In)NAs(Sb) solar cells, GaInP/GaInAsP/GaAs triple-junction solar cells, GaP/InGaAs/InGaSb triple-junction solar cells, GaInP-based multiple quantum well solar cells, and wafer-bonded InP-based four junction MJSCs [4,5,6,7,8,9]; in MJSCs, each junction is connected by a tunneling junction (TJ). In conventional III-V compound semiconductor-based MJSCs, the TJ is composed of heavily doped p-GaAs and n-GaAs. Some studies have suggested that conventional GaAs TJs can be replaced with other Al-based materials such as GaInP/AlGaAs, GaAs/AlGaAs, or AlGaAs/AlGaAs, all of which can effectively improve device performance [6,10,11]. In addition to device structure design, antireflection coatings (ARCs) are usually applied to the tops of MJSCs to reduce surface reflection and enhance incident sunlight. A strong reflection occurs at the top cell–air interface because the III-V semiconductor has an inherently high refractive index and strongly disperses wavelengths below 500 nm. The optimal thickness and refractive index of an ARC can be designed to have quarter-wavelength thickness and (*n_a_* × *n_s_*)^1/2^, where *n_a_* and *n_s_* are the refractive indices of the ambient environment and top cell, respectively. An ARC was proposed using graded-SiO_2_/TiO_2_ and a genetic algorithm to optimize the graded-SiO_2_ layer [12]. An algorithm including material dispersion can minimize reflection in a wide range of wavelengths and under varied incident angles. A triple-layer ARC composed of MgF_2_, HfO_2_, and TiO_2_ or SiC was applied to the GaAsP/Si double-junction solar cell [13]; the results indicated low reflection (<5%) within the spectrum of 390 nm to 1000 nm. A ZnO nanorod/TiO_2_ ARC was deposited on InGaP/GaAs/Ge triple-junction solar cells to increase conversion efficiency [14]; compared with bare solar cells, the InGaP/GaAs/Ge triple-junction solar cells with ZnO nanorod/TiO_2_ ARCs achieved a 30% enhancement in conversion efficiency. A ZnO nanowire ARC that incorporated an Al-doped ZnO (AZO) electrode was used in InGaP/GaAs/Ge TJ solar cells to reduce the reflectance of sunlight at the ARC–air interface [15]. The ZnO nanowire ARC combined with AZO can reduce reflectance in the short-wavelength range and achieve high conversion efficiency. Kang et al. [16] utilized coverglasses with hierarchical microstructured and subwavelength-structured surfaces to improve the absorption efficiency of InGaP/GaAs/Ge TJ solar cell modules. The short current density (J_sc_) and conversion efficiency of patterned coverglass-solar-cell modules were enhanced by 12.14% and 11.19%, respectively, compared with those of a conventional solar cell module.

Numerous researchers have reported on the surface technologies that have been used in ARCs to enhance the conversion efficiency of solar cells. ARCs can improve the lack of absorption of MJSCs under one-sun or low-concentration sunlight for terrestrial applications, and then enhance the conversion efficiency of solar cells. Popular surface technologies include a photonic crystal (PC) structure, metal/dielectric-based localized surface plasmon (LSP) structure, and textured structure. PC structures are those where a periodic variation in the refractive index occurs on the scale of the light wavelength in one or more directions [17]. An ARC with the periodic refractive index of the PC structure can diffract the waveguide mode above a certain cutoff frequency; this can improve the reflection of incident sunlight. PC-structured ARCs can be fabricated as distributed Bragg reflectors (DBRs) [18], which have a patterned surface defined by Lloyd’s mirror combined with a reactive ion etch (RIE) [19], a photolithograph-defined periodic structure [20], silicon dioxide nanospheres [21], patterned ZnO cavities [22], and polystyrene (PS)-defined AZO and SiO2 [23,24,25]. Surface plasmon coupling effects are the collective oscillations of electrons at the interface of a metal and a dielectric, and can be classified as surface plasmon polaritons at metal surfaces and the LSPs of local oscillation among isolated metallic nanostructures with resonant frequencies. LSP-coupling-affected ARCs are applied to solar cells because a solar cell with a metal surface is sheltered from the sunlight; they can be produced using silver (Ag) clusters or nanoparticles (NPs) on solar cell surfaces [26,27,28,29], Ag/indium-tin-oxide (ITO) [30], Ag/AZO [31], and ZnO/Ag/ZnO [32]. Textured surface window layers with submicron morphology on solar cells can enhance conversion efficiency by improving the absorption of broadband and divergent sunlight. Texturing surfaces by etching with diluted acid [33], ultraviolet nanoimprint lithography [34], chemical vapor deposition in high vacuum chambers [35], and crafting a rough or modified surface from a low-surface-energy material to form a superhydrophobic surface [36] are effective and commonly used processing methods.

In this study, we synthesized a silver NP/zinc oxide (Ag NPs/ZnO) thin film by using spin-coating technology. The treatment solution for the Ag NPs/ZnO thin film was composed of zinc acetate (Zn(CH_3_COO)_2_), sodium hydroxide (NaOH), and silver nitrate (AgNO_3_) aqueous solutions. Spin-coating technology offers the advantages of low cost, a large growth area, good step coverage, simple deposition equipment, and ease of preparation. The morphology of the Ag NP/ZnO thin film was observed through a field emission scanning electron microscopy (FE-SEM, JEOL, Tokyo, Japan), and the film’s Ag content was determined using energy-dispersive X-ray spectroscopy (EDS) (JSM-7500F, JEOL, Tokyo, Japan). The crystalline characteristics of the Ag NP/ZnO thin film were characterized by X-ray diffraction (XRD) patterns using an advanced diffractometer (Bruker D8, Billerica, MA, USA) equipped with CuKa (λ = 0.154 nm). The root mean square (RMS) roughness of the Ag NP/ZnO film was analyzed using atomic force microscopy (AFM) (D13100, Digital instruments Veeco Metrology Group, Plainview, NY, USA). Finally, the Ag NPs/ZnO thin film was deposited on SiN_x_-coated GaInP/GaAs/Ge solar cells. The current density versus voltage (JV) characteristics for completed solar cell chips were measured using a solar simulator with an Xe lamp light source calibrated to a one-sun condition. During JV measurements, the ambient temperature was controlled using a temperature control stage (STC200, Instec, Boulder, CO, USA). The Ag NPs/ZnO thin film with SiN_x_ was used as a hybrid ARC to reduce the reflection of sunlight. The conversion efficiency and short current density of GaInP/GaAs/Ge solar cells with the optimal hybrid ARC constituted a substantial improvement over GaInP/GaAs/Ge solar cells with SiN_x_ ARCs.

## 2. Results and Discussion

The Zn(CH_3_COO)_2_ aqueous solution composed of Zn^2+^ and CH_3_COO^−^ ions was mixed with the NaOH aqueous solution containing Na^+^ and OH^−^ ions to form a ZnO treatment solution. Ions in the ZnO treatment solution reacted to form a ZnO thin film on the silicon nitride-coated (SiN_x_) glass (SNG) substrate through the following reaction:

Zn(CH_3_COO)_2_ + 2 NaOH ⇄ Zn^2+^ + 2 CH_3_COONa + 2 OH^−^, (1)

Zn^2+^ + 4 OH^−^ ⇄ Zn(OH)_4_^2−^, (2)

Zn(OH)_4_^2−^ ⇄ Zn(OH)_2_ + 2 OH^−^, (3)

Zn(OH)_2_ ⇄ 2 H^+^ + ZnO_2_^2−^, (4)

Zn^2+^ + 2 ZnO_2_^2−^ ⇄ ZnO, (5)

AgNO_3_ added to a ZnO treatment solution, as an Ag NP/ZnO treatment solution can thermally decompose into Ag NPs, gaseous oxynitride, and oxygen during the post-annealing process at 200 °C, as indicated in the following reaction [28]:

AgNO_3_ ⇄ 2 Ag +2NO_2_ +O_2_, (6)

Consequently, the Ag NP/ZnO thin film was obtained on the SNG substrate after spin-coating and the 200 °C post-annealing process.

Table 1 lists the O, Zn, and Ag contents of ZnO and Ag NP/ZnO thin films measured using EDS at AgNO_3_ concentrations of 0.005 M, 0.008 M, 0.02 M, and 0.05 M. The Zn^2+^, Zn(OH)_4_^2−^, Zn(OH)_2_, and ZnO_2_^2−^ ions in the ZnO treatment solutions reacted to form a ZnO thin film through the process expressed in Equations (2)–(5). The Ag content of the Ag NP/ZnO thin film increased when the AgNO_3_ concentration rose from 0.005 M to 0.02 M, because large numbers of Ag NPs developed in the Ag NP/ZnO thin film, according to Equation (6). Furthermore, the Ag content in the Ag NP/ZnO thin film increased with the AgNO_3_ concentration, but the Zn and O contents remained constant, implying the formation of Ag NPs rather than AgO or Ag_2_O in the Ag NP/ZnO thin film; AgO and Ag_2_O may form in a solution of AgNO_3_ mixed with NaOH [37].

**Figure 1:**
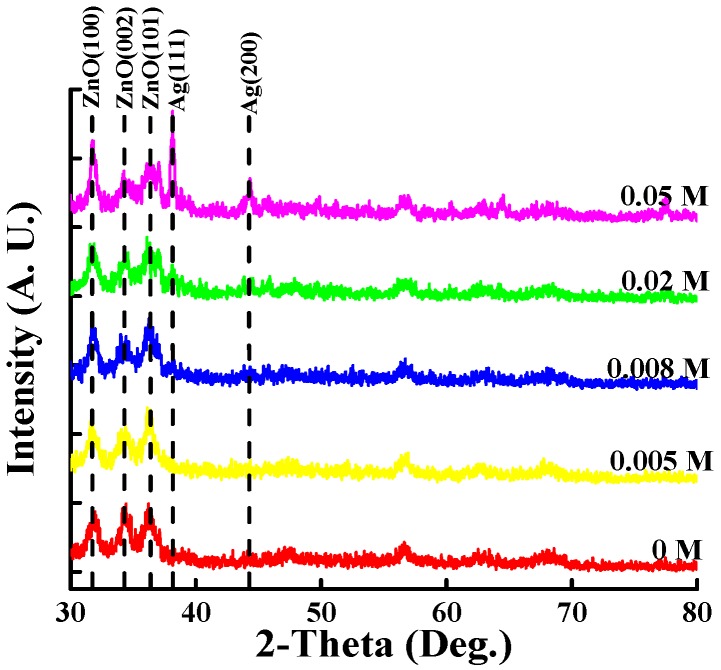
X-ray diffraction (XRD) spectra of spin-coated ZnO thin films and Ag NP/ZnO thin films with various AgNO_3_ concentrations.

To study the crystallization of Ag NPs on ZnO thin films, the XRD spectra of the Ag NP/ZnO thin films at various AgNO_3_ concentrations were measured, and are plotted in Figure 1. A preferential (002) peak (34.4°) alongside a (101) peak (36.2°) and (100) peak (31.8°) was found in the spin-coated ZnO thin film, thereby indicating a hexagonal wurtzite structure and a polycrystalline nature. Similar XRD patterns were observed in spin-coated Ag NP/ZnO thin films with extra peaks of face-center-cubic crystal Ag, namely a (111) peak (38.1°) and (200) peak (44.3°). As depicted in Figure 1, the diffraction intensity of the Ag (111) and Ag (200) peaks gradually increased when the AgNO_3_ concentrations rose from 0 M to 0.05 M because of the increase in Ag content in the Ag NP/ZnO treatment solution, as indicated in Equation (6). Furthermore, no diffraction peak for Ag oxides such as AgO or Ag_2_O occurred (see Figure 1) because of decomposition of Ag oxide to Ag and O_2_ during the 200 °C post-annealing process [32]. Ag NP reduction at an AgNO_3_ concentration of 0.005 M is rare in an Ag NP/ZnO treatment solution, because the low AgNO_3_ concentration engenders low diffraction intensity and a wide pattern of Ag (111) and (200) peaks. As the AgNO_3_ concentration increased from 0.008 M to 0.05 M, a large quantity of Ag NPs formed in the treatment solution; consequently, these small-sized NPs aggregated at the surface or interfaced between ZnO grains, and gathered to a large grain size of Ag NPs during the post-annealing process, resulting in an enhanced diffraction intensity and narrow pattern of Ag (111) and Ag (200) peaks. In addition, the intensity of the ZnO-(100)-indexed diffraction peak increased, whereas that of the ZnO-(002)-indexed diffraction peak decreased with the rising AgNO_3_ concentration; this was attributed to the (002)-orientated ZnO thin films being destroyed or bended by the large numbers of Ag NPs at a high AgNO_3_ concentration [38].

**Figure 2:**
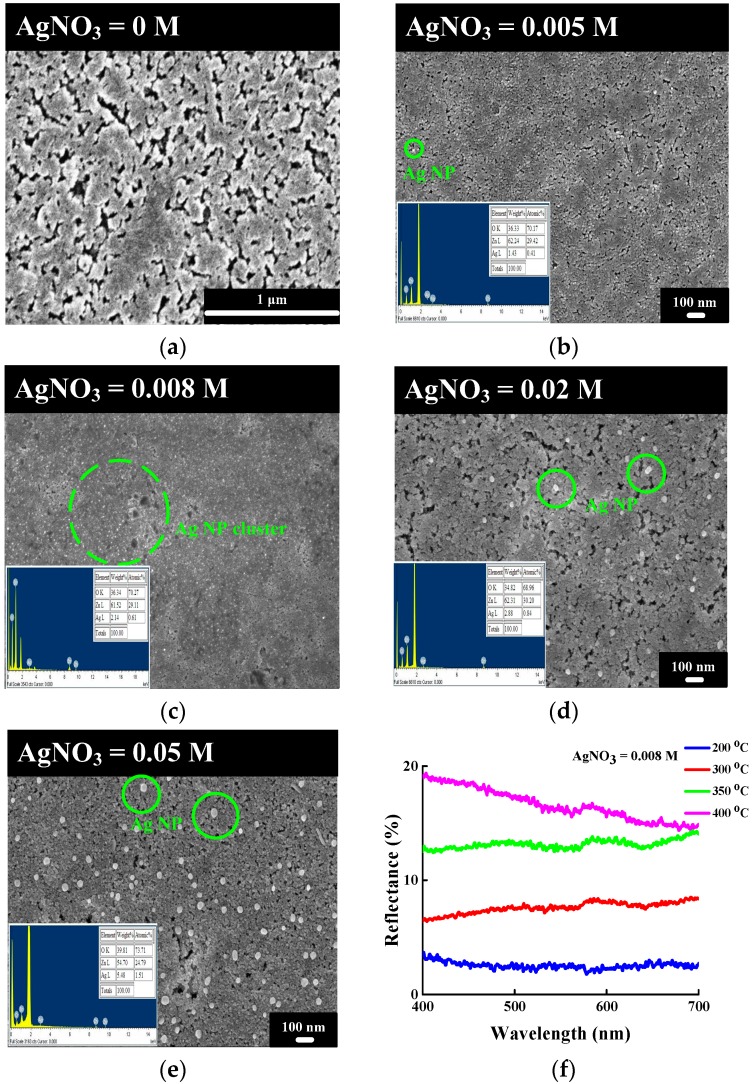
SEM images for (**a**) spin-coated ZnO and silver nanoparticle/zinc oxide (Ag NP/ZnO) grown at AgNO_3_ concentrations of (**b**) 0.005 M, (**c**) 0.008 M, (**d**) 0.02 M, and (**e**) 0.05 M; the inset of (**b**–**e**) are the energy-dispersive X-ray spectroscopy (EDS) spectra. (**f**) is the reflectivity of Ag NP/ZnO/SiN_x_ hybrid thin films grown at various post-annealing temperatures.

The surface morphologies of the ZnO and Ag NP/ZnO thin films were examined based on scanning electron microscopy (SEM) images. Figure 2 depicts top-view images of the ZnO and Ag NP/ZnO thin films at various AgNO_3_ concentrations on SNG substrates. According to Equations (1)–(5), the ZnO thin films without Ag NPs were able to develop, and then formed on the SNG substrate after spin-coating and the post-annealing process; the surface morphology of the ZnO thin film was textured and rough, as depicted in Figure 2a. A textured ZnO thin film can serve as an ARC to reduce reflectivity in the GaInP/GaAs/Ge triple-junction solar cell [39]. Figure 2b depicts the Ag NP/ZnO thin film prepared with an AgNO_3_ concentration of 0.005 M. A small grain size and rare distribution of Ag NP were observed and are depicted in Figure 2b; the rare distribution was attributable to the small number of Ag NPs in the treatment solution, and the small grain size was attributed to the incomplete gathering of Ag NPs under a post-annealing temperature of 200 °C; a rare distribution of Ag NPs cannot effectively reduce the reflection of sunlight for solar cell applications [27,39,40]. Although the grain size and distribution of Ag NPs on Ag NP/ZnO thin films can be improved by increasing the post-annealing temperature, a high annealing temperature will rise the reflectivity of Ag NP/ZnO/SiN_x_ hybrid thin films, as shown in Figure 2f, possibly due to the larger grain sizes of the Ag NPs. In order to achieve a small interval between two Ag NPs and large-grain-sized Ag NP on Ag NP/ZnO thin films without raising the post-annealing temperature, an Ag NP/ZnO treatment solution with a high AgNO_3_ concentration can be used to deposit Ag NP/ZnO on thin films. Figure 2c–e depict top-view SEM images of Ag NP/ZnO thin films on an SNG substrate prepared with AgNO_3_ concentrations of 0.008 M, 0.02 M, and 0.05 M. When the AgNO_3_ concentration increased to 0.008 M, a small interval between two Ag NPs on Ag NP/ZnO thin films was achieved after the post-annealing process, because of the large number of Ag NPs in the Ag NP/ZnO treatment solution. However, the distribution and grain size of the Ag NPs on the Ag NP/ZnO thin films depicted in Figure 2d,e were rarer and larger than those depicted in Figure 2c. A high (>0.008 M) AgNO_3_ concentration in the Ag NP/ZnO treatment solution led to a large quantity of Ag NPs and short spaces between Ag NPs; these factors can enhance the gathering of Ag NPs on Ag NP/ZnO thin films during the post-annealing process. Consequently, the interval between two Ag NPs and the grain size of the Ag NPs on the Ag NP/ZnO thin films grown at AgNO_3_ concentrations of 0.02 M and 0.05 M were larger than those grown with an AgNO_3_ concentration of 0.008 M.

**Figure 3:**
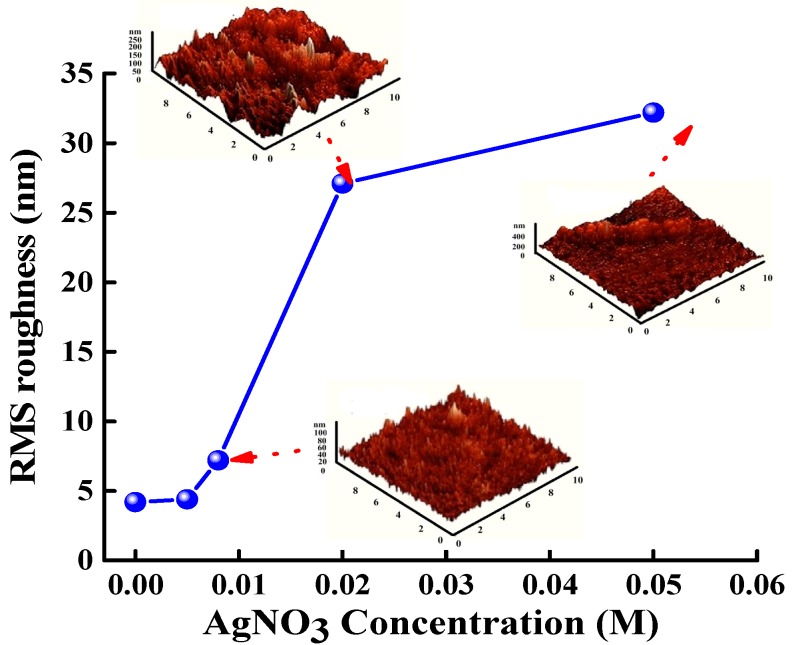
Root mean square (RMS) roughness of Ag NP/ZnO thin films as a function of AgNO_3_ concentration; the insets show atomic force microscopy (AFM) images of Ag NP/ZnO thin films prepared with AgNO_3_ concentrations of 0.008 M, 0.02 M, and 0.05 M.

Figure 3 depicts the RMS roughness of the Ag NP/ZnO thin film as a function of Ag NO_3_ concentration; the insets of Figure 3 present AFM images of the Ag NP/ZnO thin films prepared with AgNO_3_ concentrations of 0.008 M, 0.02 M, and 0.05 M. The RMS roughness of the Ag NP/ZnO thin films increased when the AgNO_3_ concentration rose from 0.005 M to 0.008 M, and increased rapidly for the Ag NP/ZnO thin films grown at AgNO_3_ concentrations above 0.008 M. A large quantity of Ag NPs were present in the Ag NP/ZnO treatment solution with a high AgNO_3_ concentration, according to Equation (6), and these Ag NPs ripened to a large grain size during the post-annealing process, which led to high RMS roughness. The AFM images of the Ag NP/ZnO thin films indicate a weak textured surface morphology at an AgNO_3_ concentration of 0.008 M, but strong textured surface morphologies at AgNO_3_ concentrations of 0.02 M and 0.05 M. These textured Ag NP/ZnO thin films can be used as window layers of solar cells to enhance light trapping.

**Figure 4:**
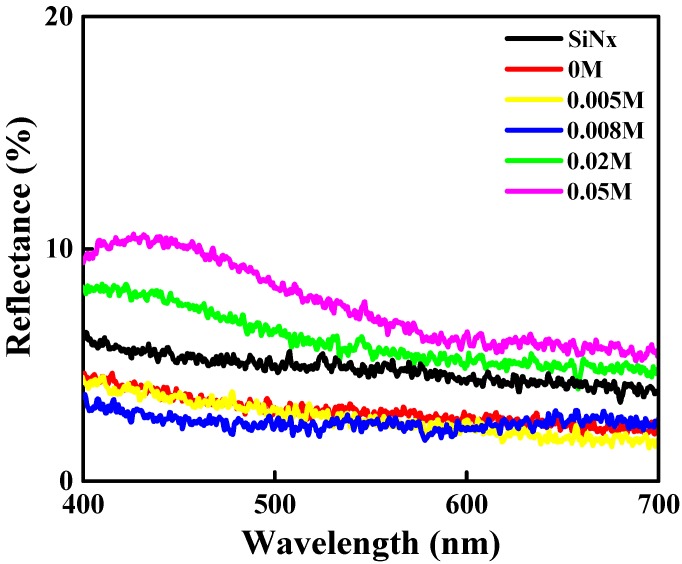
Reflectivity of the Ag NP/ZnO/SiN_x_ hybrid thin films grown at various AgNO_3_ concentrations as a function of wavelengths of 400–700 nm.

Figure 4 depicts the reflectivity of the Ag NP/ZnO/SiN_x_ hybrid thin film (named “hybrid ARC”) grown at varied AgNO_3_ concentrations as a function of wavelength over 400–700 nm. The average reflectivity of the hybrid ARCs grown at AgNO_3_ concentrations of 0.005 M, 0.008 M, 0.02 M, and 0.05 M were 2.99%, 2.67%, 2.53%, 5.99%, and 7.47%, respectively, all of which were lower than the reflectivity of the SiN_x_ ARC (approximately 9.2%) obtained in our previous study [39]. The effective refractive index (n_eff_) of the hybrid ARC is approximately 1.87, and can be calculated using the following equation:

(7) $$n_{\mathrm{eff}}=\frac{2}{\frac{1}{n_{\mathrm{ZnO}}}+\frac{1}{n_{\mathrm{SiNx}}}},$$

where n_ZnO_ (1.76) and n_SiNx_ (2.0) are the refractive indices of Ag NP/ZnO and SiN_x_ thin films, respectively. The calculated refractive index of the ARC (n_ARC_) is approximately 1.84, which can be calculated from n_ARC_ = (1 × n_GaAs_)^1/2^, where n_GaAs_ is the refractive index of GaAs. The refractive index of the hybrid ARC (1.87) is closer to 1.84 than is SiN_x_ (2.0), leading to low reflectivity. The amplitude of reflectance (r_APZN_) and reflectivity (R_APZN_) between air and Ag NP/ZnO thin films are provided in [41] and expressed as follows:

(8) $$r_{\mathrm{APZN}}=\frac{n_{\mathrm{ZnO}}-1}{n_{\mathrm{ZnO}}+1}\exp\left[-\frac{1}{2}\left(\frac{{4\pi \sigma n}_{\mathrm{ZnO}}}{\lambda }\right)\right],$$

(9) $$R_{\mathrm{APZN}}={\left|\frac{n_{\mathrm{ZnO}}-1}{n_{\mathrm{ZnO}}+1}\exp\left[-\frac{1}{2}\left(\frac{{4\pi \sigma n}_{\mathrm{ZnO}}}{\lambda }\right)\right]\right|}^{2},$$

where σ is the RMS roughness of the Ag NP/ZnO thin film, and λ is the wavelength of light in a vacuum. The reflectivity of the Ag NP/ZnO thin film decreased when the AgNO_3_ concentration increased from 0 M to 0.008 M; this was attributable to the increasing RMS roughness depicted in Figure 3. However, the reflectivity of the Ag NP/ZnO thin films increased when the AgNO_3_ concentration increased from 0.02 M to 0.05 M; this was attributable to the potent light scattering that resulted from the large grain size of Ag NPs evident in the SEM and AFM images.

**Figure 5:**
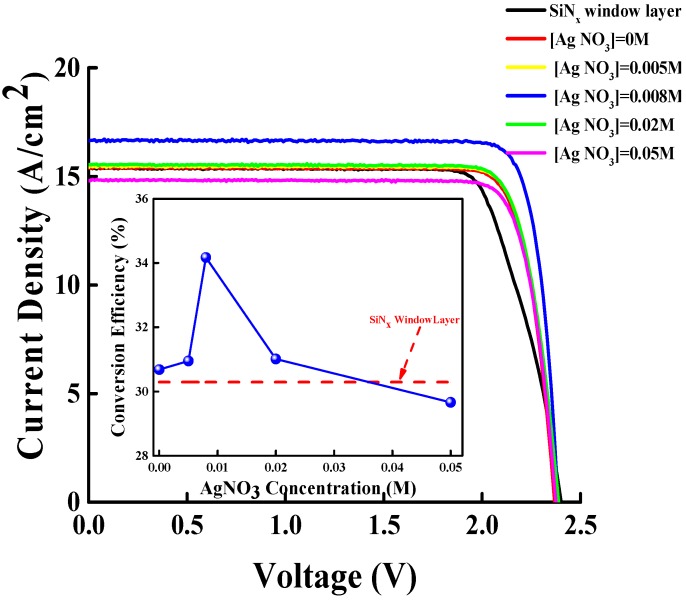
Current density versus voltage (JV) characteristics of GaInP/(In)GaAs/Ge solar cells with the Ag NP/ZnO/SiN_x_ hybrid antireflection coatings (ARCs) grown at AgNO_3_ concentrations of 0.005 M, 0.008 M, 0.02 M, and 0.05 M; the inset indicates the conversion efficiency of GaInP/(In)GaAs/Ge solar cells with related ARCs.

Figure 5 depicts the JV characteristics of the GaInP/(In)GaAs/Ge solar cells with the SiN_x_ ARC and the Ag NP/ZnO/SiN_x_ hybrid ARCs grown at AgNO_3_ concentrations of 0 M, 0.005 M, 0.008 M, 0.02 M, and 0.05 M. The inset of Figure 5 indicates the conversion efficiency of the GaInP/(In)GaAs/Ge solar cells with related ARCs. The short-circuit current density of the GaInP/(In)GaAs/Ge solar cells with SiN_x_ ARC and hybrid ARCs grown at AgNO_3_ concentrations of 0 M, 0.005 M, and 0.008 M were 15.37 mA/cm^2^, 15.39 mA/cm^2^, 15.43 mA/cm^2^, and 16.63 mA/cm^2^. A high light-trapping effect achieved through a reduction in the surface reflectivity of GaInP/(In)GaAs/Ge solar cells is required to obtain a high short-circuit current density and conversion. Compared with the SiN_x_ ARC, the hybrid ARC with a lower surface reflectivity enhanced the short-circuit current density of the GaInP/(In)GaAs/Ge solar cell because of the high transmitting intensity of sunlight into the solar cell. Moreover, the short-circuit current density of the GaInP/(In)GaAs/Ge solar cell with an Ag NP/ZnO ARC grown at an AgNO_3_ concentration of 0.008 M noticeably increased; this was attributable to the low surface reflectivity and high light trapping. The short-circuit current densities of the GaInP/(In)GaAs/Ge solar cells with Ag NP/ZnO ARCs grown at AgNO_3_ concentrations of 0.02 M and 0.05 M decreased to 15.54 mA/cm^2^and 14.79 mA/cm^2^, respectively; these findings were attributable to the high surface reflectivity and light scattering that resulted from the large grain size of the Ag NPs. In this study, the conversion efficiency of a solar cell depended on the short-circuit current density because the open-circuit voltage and fill factor were fairly constant. As depicted in the inset of Figure 5, the GaInP/(In)GaAs/Ge solar cell with an Ag NP/ZnO ARC grown at an AgNO_3_ concentration of 0.008 M demonstrated a maximum conversion efficiency of 34.17% because of the high short-circuit current density.

Spin-coating is a method for synthesizing Ag NP/ZnO thin films through a chemical reaction in an aqueous solution at room temperature. According to the XRD (Figure 1) and SEM (Figure 2) images, and EDS analysis (Table 1), the Ag content and Ag NP grain size of Ag NP/ZnO/SiN_x_ hybrid ARCs depend on the AgNO_3_ concentration in treatment solution; Ag NPs in the treatment solution are in the form of clusters on the surface of ZnO thin films or at the interfaces between ZnO grains during the post-annealing process. The RMS roughness of Ag NP/ZnO/SiN_x_ hybrid ARCs related to the grain size of Ag NPs determines the surface reflectivity; the reflectivity of Ag NP/ZnO/SiN_x_ hybrid ARCs can be adjusted by AgNO_3_ concentration. Table 2 shows the measured short-circuit current density, open-circuit voltage, fill factor, and conversion efficiency of GaInP/(In)GaAs/Ge solar cells with varied ARCs. The highest conversion efficiency of 34.17% can be observed in Table 2 with the textured Ag NP/ZnO/SiN_x_ hybrid ARC proposed by an AgNO_3_ concentration of 0.008 M. The RMS roughness of an Ag NP/ZnO/SiN_x_ hybrid ARC below 7.2 nm (with an AgNO_3_ concentration of 0.005 M) cannot effectively reduce the surface reflection, as indicated in Equation (9); the RMS roughness of the Ag NP/ZnO/SiN_x_ hybrid ARC, which is higher than 7.2 nm (with an AgNO_3_ concentration of 0.02 M or 0.05 M), shows a low reflectivity; the Ag NP with large grain sizes causes high reflection and light scattering.

## 3. Experimental Details

### 3.1. Deposition of the Ag NP/ZnO Thin Film

The Ag NP/ZnO treatment solution consisted of zinc acetate (Zn(CH_3_COO)_2_), sodium hydroxide (NaOH), and silver nitrate (AgNO_3_) aqueous solutions. Zn(CH_3_COO)_2_ and AgNO_3_ were used as the raw materials for preparing zinc oxide (ZnO) and silver NPs (Ag NPs), and NaOH was used as a reductant. Since the solubility levels of Zn(CH_3_COO)_2_, NaOH, and AgNO_3_ powder in de-ionized (DI) water differed, these powders were dissolved in DI water individually to form aqueous solutions with 0.007 M, 1 M, and 0.005–0.1 M concentrations under room temperature. These solutions were then mixed and stirred uniformly to form an Ag NP/ZnO treatment solution at room temperature. Before the Ag NP/ZnO treatment solution was spread on the silicon nitride-coated (SiN_x_) glass (SNG) substrate and the GaInP/GaAs/Ge solar cell with an SiN_x_ layer, the surfaces of these substrates were treated using an oxygen plasma to obtain a hydrophilic surface. The substrates were baked on a hot plate at 100 °C for three minutes for dewatering after the Ag NP/ZnO treatment solution had been spread on the substrates via the spin-coating process. Finally, the dewatered SNG substrate and GaInP/GaAs/Ge solar cell with an SiN_x_ layer were coated with the Ag NP/ZnO treatment solution and treated through a post-annealing process at 200 °C for 1 h in an N_2_-ambient furnace to form the Ag NP/ZnO thin film.

### 3.2. Fabrication of GaInP/GaAs/Ge Solar Cells with an Ag NP/ZnO Window Layer

GaInP and (In)GaAs were grown on 150-μm thick p-type Ge substrate through metal–organic chemical vapor deposition (MOCVD). The TJs used to link the Ge substrate, InGaAs, and GaInP subcells were heavily doped with n-GaAs/p-GaAs and p-AlGaAs/n-GaInP. An alloy composed of Au/Zn/Ag/Au was coated onto the back of the p-Ge substrate as a p-type contact metal. The wafer was successively patterned using a standard photolithographic process to define the n-contact region through partial exposure of n-InGaAs. An alloy composed of AuGe/Ni/Au was used as an n-type contact metal in the n-InGaAs contact region. Finally, an Si_3_N_4_ (n = 2.0) grown through plasma-enhanced chemical vapor deposition and an Ag NP/ZnO thin film were coated onto n-AlInP, which was defined through partially etched n-InGaAs.

## 4. Conclusions

We proposed an Ag NP/ZnO thin film on an SNG substrate and a GaInP/(Al)GaAs/Ge solar cell created using spin-coating technology. The grain size and content of Ag on the Ag NP/ZnO thin film depended on the AgNO_3_ concentration in the Ag NP/ZnO treatment solution. An optimal hybrid ARC comprising SiN_x_ and Ag NP/ZnO thin film grown with an Ag NP/ZnO treatment solution contained 0.0008 M AgNO_3_, 0.007 M Zn(CH_3_COO)_2_, and 1 M NaOH; following the 200 °C post-annealing process; this ARC exhibited a lower average reflectivity (2.53%) over wavelengths of 400–700 nm compared with the conventional SiN_x_ ARC. This finding was attributable to the textured surface, as determined by the grain size of the Ag NPs, the ZnO surface morphology, and a suitable effective refractive index constructed of SiN_x_ and Ag NP/ZnO. GaInP/(Al)GaAs/Ge solar cells incorporating the optimal hybrid ARC demonstrated a high conversion efficiency rate of 34.1%, with V_oc_ = 2.39 V, J_sc_ = 16.63 mA/cm^2^, and fill factor = 86.1%. Furthermore, well scale-defined and uniform-distributed Ag NPs are required in an Ag NP/ZnO/SiN_x_ hybrid ARC to reduce the reflectivity of GaInP/(Al)GaAs/Ge solar cells for industrial-scale application. An adjusted spinner speed and controlled drop volume of treatment solution would be the method for fulfilling the aforementioned requirements.

## Figures and Tables

**Table 1:** O, Zn, and Ag contents of ZnO and Ag nanoparticle (NP)/ZnO thin films.

AgNO_3_ Concentration (M)	O Content (at %)	Zn Content (at %)	Ag Content (at %)
0	70.02	29.98	0
0.005	70.2	29.4	0.4
0.008	70.3	29.1	0.61
0.02	69	30.2	0.8
0.05	73.7	24.8	1.51

**Table 2:** Short-circuit current density (J_SC_), open-circuit voltage (V_OC_), fill factor, and conversion efficiency of GaInP/(In)GaAs/Ge solar cells with related ARCs (AM 0, 1 sun).

ARC	J_SC_ (mA/cm^2^)	V_OC_ (V)	Fill Factor (%)	Conversion Efficiency (%)
**SiN_x_**	15.37	2.4	78.8	30.2
**Ag NP (0 M)/ZnO/SiN_x_**	15.39	2.37	83.1	30.26
**Ag NP (0.005 M)/ZnO/SiN_x_**	15.43	2.38	84.2	30.95
**Ag NP (0.008 M)/ZnO/SiN_x_**	16.63	2.39	86.1	34.17
**Ag NP (0.02 M)/ZnO/SiN_x_**	15.54	2.39	83.5	31.01
**Ag NP (0.05 M)/ZnO/SiN_x_**	14.79	2.38	84.4	29.76
